# Does Samoa Have Adequate Policies to Reduce Obesity and Obesity-Related Disease?

**DOI:** 10.7759/cureus.23239

**Published:** 2022-03-16

**Authors:** Viali Lameko

**Affiliations:** 1 Internal Medicine, Oceania University of Medicine, Apia, WSM

**Keywords:** samoa, obesity, risk factors, behavior, nutrition

## Abstract

In obesity, abnormal or excess body fat (adiposity) impairs health, increases the risk of long-term medical complications, and results in decreased lifespan. There are many long-term medical complications to consider, such as heart disease, cancer, stroke, type 2 diabetes mellitus, and non-alcoholic fatty liver disease, among others. These are all difficult and expensive conditions to treat in a small developing country like Samoa. The prevalence of obesity in Samoa has steadily increased since the 1960s, and today, 53% of Samoa’s adult population are obese. People living in the small island country of Samoa, with a total population of fewer than 200 thousand people, are one of the most obese people in the world today. Factors that have contributed to obesity over the previous four decades include urbanization, the dependence on a dietary transition from one based on fresh local food to highly processed imported food, poverty, as well as probable genetic predisposition. Efforts by the Samoa Ministry of Health and partners to reverse the trend of obesity have mainly relied on behavior change strategies since the 1970s, with no clinical evidence of success. This study critically reviews current policies and strategic plans in Samoa to reduce the obesity problem and proposes that because of the contributing structural factors, the health sector alone cannot achieve its objective without matching multi-sectorial government initiatives.

## Introduction and background

Obesity is a complex chronic disease in which abnormal or excess body fat (adiposity) impairs health, increases the risk of long-term medical complications, and reduces lifespan [1]. Long-term medical complications include heart disease, cancer, stroke, type 2 diabetes mellitus, and non-alcoholic fatty liver disease, among others [2]. The world’s population is increasingly becoming overweight or obese, and the prevalence of obesity is associated with rising rates of non-communicable diseases (NCDs). In 1977, Zimmet et al. warned of a “diabesity” epidemic (obesity and type 2 diabetes) based on studies of small Pacific Island states, forecasting that it was likely to be the biggest epidemic in human history [3]. The accuracy of this prediction is confirmed by recent findings for Samoa where approximately 53.8% of adults; 25 years to 64 years old are obese [4]. Over the period of 1978-2013, in a population of approximately 200,000 Polynesian people, the prevalence of obesity increased from 27.7% to 53.1% in men (2.3% per five years) and 44.4% to 76.7% in women (4.5% per five years) [5]. Obesity prevalence in 2020 is projected to reach 59.0% among men and 81.0% among women, making obesity the leading cause of disability in Samoa [5]. Furthermore, over 60% of the adult population is obese in other small Pacific Island states and territories; Nauru, American Samoa, Cook Islands, Niue, Tokelau, and Tonga [6]. Furthermore, Samoans, like other Polynesians, may have a genetic predisposition to diseases associated with obesity such as diabetes and gout [7].

The prevalence of obesity in Samoa and other Pacific Island countries has steadily increased since the 1960s despite more than forty years of behavior change focused public health programs advising Pacific Islanders to consume more fresh local foods and drinks having high sugar content and consume less imported foods and drinks having high sugar contents and other refined carbohydrates, salt, and fat [8]. As in other developing countries, NCDs in Samoa are a serious challenge because rising health care costs impose increasing financial burdens on the government [8]. Hence, the prevalence of adult obesity in Samoa and some other small Pacific Island countries place a heavy burden on health resources. To tackle the structural contributors to rising obesity, multi-sectorial policies are needed. This article addressed the question of what is really driving obesity rates in Samoa, and whether the policy responses are adequate.

## Review

Traditional diet and lifestyle of Samoa and Pacific people

Most studies of the high prevalence of obesity and NCDs in small Pacific Island states and territories locate the causes primarily in dietary and lifestyle changes [5-8]. A revealing study of links between food availability, food prices, and obesity in Samoa found that the total energy available from food increased by 47%, with more than 900 extra calories available per capita per day between 1961 and 2007 [9]. A consequence has been a rise of 18% in mean body mass index (BMI) for men and women aged 35-44 years old also recorded between 1980 and 2007. In that period, the traditional Samoan diet of staple vegetable root and tree crops, coconut, fish and other seafood, native birds, and more rarely, pork and chicken have largely been replaced by imported processed foods such as canned and brined beef, canned fish, mutton flaps, factory-farmed chicken, turkey tails, heavily sweetened beverages, bread and other flour-baked goods, and rice, to name a few [9]. There was virtually no sugar in the pre-colonial diet of Samoans which was high in complex carbohydrates, in contrast to the present day diet patterns where sugar is consumed in large amounts [10,11]. Not only have diets and eating habits changed but Samoans have become less physically active [4].

Samoan government policies and initiatives: impacts on obesity

In 2006, the Government of Samoa decided to change the roads from left-hand to right-hand drive [12]. One of the arguments for doing this was that it would make motor vehicles cheaper so that farmers in Samoa could import cars or trucks bought by their relatives living in New Zealand and Australia to help them develop their plantations and farms and thereby increase production, sales, and export of crops. This policy has resulted in a dramatic increase in the number of privately owned vehicles. However, the downside of this change has been reduced physical activity among the population contributing to obesity. Today farmers and their family members, who used to walk to and from their plantations, carrying their crops in baskets on their shoulders, now use motor vehicles for this purpose. Furthermore, most people walk less but drive even short distances from their homes to shops, churches, and schools for the children. In rural Samoa, it is now rare to see fishermen using the traditional canoes for fishing because the canoes have been replaced by motorized boats.

Drivers of the obesity problem in Samoa

The prevalence of obesity in Samoa and elsewhere, as is recognized by the World Health Organization, is a result of choices made by individual consumers [13]. However, the structural factors in the Samoa context, include urbanization, preference for wage employment over farming, migration, remittances, poverty, and cheap imported food [13]. Imported food is often cheaper than fresh local food, for example, imported factory-farmed chicken parts are much cheaper than locally caught fish and other seafood [9]. A serving of rice or white bread is cheaper than the same amount of taro, which was the staple food of all Samoans. The prices of soft drinks are almost the same price as a bottle of water of the same volume, and a green drinking coconut is only slightly cheaper. 

Other drivers of obesity in Samoa and other small island countries in the Pacific, also identified by WHO, are global forces, including international free trade and expansion of markets. Statistics for 2018-2019 show that Samoa exports goods worth USD$70m and imports goods worth USD$510m, of which food is a significant component. The latest figures on the value of personal remittances received in Samoa is USD$141,547,900 [14]. Changing aspirations have changed the cultural and social structure; driving people away from subsistence farming to wage-earning and petty trading, and population movements from rural villages to urban areas with a more sedentary lifestyle. Evidence of this trend is demonstrated in a recent study showing that there has been no significant growth in population and economic activity in four representative villages over the past 50 years, due to migration overseas and to urban settlements [15]. 

Current policies and government strategies

The Government of Samoa, like many other small island countries in the Pacific, recognizes the high national costs of NCDs and obesity leading to the development of National NCD Control policies for 2010-2015 and 2018-2023, and the Samoa National Health Promotion Policy 2010-2015 [6]. The National Food and Nutrition Policy 2013-2018 is being currently being reviewed to prepare a new and updated nutrition policy for 2021-2025 [16]. The NCD policy has five broad key strategic areas: (i) governance, leadership and partnership in health; (ii) health promotion, advocacy, and risk reduction; (ii) health system strengthening to address NCDs; (ii) surveillance, monitoring and evaluation; and (ii) climate change and NCDs. It aligns with other national policies, such as Samoa’s Strategy for Development (SDS) which has an overriding vision of “attaining an improved quality of life for all Samoans” [17]. It also aligns with regional and world plans and declarations, such as the World Health Organization Global Action Plan for NCD, 2013-2020, and others [18].

Not only have diets and lifestyles changed in Samoa but so have health delivery systems. The public health system in Samoa was once community-based but is now hospital-based. Between 1930 and 1980, it relied on hundreds of village women’s committees throughout Samoa to promote disease prevention, each serviced by a district public health nurse who met with the committees monthly to hold maternal and child health clinics and sanitation inspections. By the 1970s, monthly village health talks by the district nurses included advice on nutrition using health messages formulated for the Pacific Islands region by the South Pacific Commission (now the Secretariat of the Pacific Community). However, in the 1980s, the women’s committees were de-linked from the public health nursing services in the Ministry of Health to support a broacher agenda for "women in development" and the policy changes in health services meant that nurses no longer went out to villages (except in the case of special programs), instead people in villages sought advice and treatment from the nearest health center or district hospital, or if they had the means to do so, went to the outpatient clinics at the main hospitals on Upolu and Savaii [19]. At the same time, the distribution of the population had begun to change significantly, today only about 60% of the population live in traditional villages; urban settlements have grown around the town of Apia with no effective local government [20].

The public health system in Samoa is now fully under the Samoa Ministry of Health, which is responsible for all the public health services and clinical facilities in the country. These facilities include the main referral hospital located in the capital city of Apia and seven peripheral district hospitals located on the two main islands of Upolu and Savaii. In early 2020, as part of the coronavirus disease 2019 (COVID-19) national response, a resident medical officer was appointed to each district hospital. There are two medical laboratories and two public pharmaceutical dispensaries, one operated at the main hospital in Apia, and one at the main hospital on Savaii Island.

Public expenditure on health services in Samoa is one of the highest among the small island countries in the Pacific [21]. Between 80% and 90% of the total health care in Samoa is government-financed, with between 10% and 20% payments from private and out-of-pocket expenditure. Aid to the health sector is mostly provided by New Zealand; other donors include China, Australia, and Japan, as well as technical assistance from the WHO and other United Nations agencies. Despite a continued burden of infectious disease, of the total public health expenditure, the clinical care and treatment of non-communicable diseases (NCDs) accounts for over 40% of expenditure, and in addition, the cost of operating the hemodialysis services in Samoa by the Samoa Kidney Foundation is estimated at $96,715/patient/year (local currency) or USD 38,686 [21].

Samoa’s health policy is now re-emphasizing community-based interventions such as school programs to promote physical education and after-school sport, and engagement with village women’s committee to support screening of NCD risk factors among members of the community and referring those who are at high risk for further medical treatment. But for the most part, the focus is on the promotion of behavior change to reduce the prevalence of obesity through social marketing explaining risk factors and advocating healthy diets and lifestyles. Health messages are disseminated through community seminars and workshops about healthy eating, physical exercise and vegetable gardening using social media and television, radio, and newspapers. This follows the same awareness and behavior change approach, albeit using more modern media, that has been used since the 1970s when it was first promoted by the South Pacific Commission (now the Secretariat of the Pacific Community), WHO, United Nations Children's Fund (UNICEF), and other agencies [3].

Although Samoa’s policy advocates a multi-sector approach to reducing NCDs, it offers no details of how the structural challenges referred to above might be overcome. This is the responsibility of other ministries outside of the Ministry of Health. In addition, the recent health-sponsored television commercials do not mention the importance of reducing the consumption of sugar in their diet but focus only on performing physical exercise and eating vegetables. Shown on prime time TV, they are often followed by advertisements for sweet drinks and other sugary products by local manufacturers and retailers. Given the evident historical failure of behavior change approaches, there are questions to be asked about the degree to which behavior and personal choices are contributing factors to the development of overweight and obesity in Samoa and the rest of the world today. 

Obesity is a political as well as a policy issue. If the Government of Samoa is to offset the rising cost of treating NCDs, it will have to adopt a more active multi-sectoral approach which could include laws, taxes, tariffs, and subsidies to increase consumption of fresh local food and decrease consumption of imported food. But such measures will be politically unpopular, especially with local importers, wholesalers, retailers, and manufacturers. Unless carefully applied, such measures may make food too expensive for public consumption. Local manufacturers produce beer, soft drinks, ice cream, and various snack foods made from ingredients imported via global supply chains. Local merchants import hundreds of similar inexpensive products. The food industry had opposed any increase in taxes on their products, in the recent past. For instance, when levies were imposed on sugar-sweetened beverages, both the local producers and importers of soft drinks in Samoa argued that the consumers are the ones who would carry the burden of the increased taxes. They persistently lobbied for the removal of the excise taxes. In the past policy actions aiming to restrict the importing of foods thought to be harmful, such as turkey tails and mutton flaps, have been thwarted by the terms of international trade agreements [22].

Many new measures are needed. There should be a ban on television advertisements promoting sugar-sweetened products, especially those targeting children during prime time. Similarly, Samoa should draw on the successful measure used to reduce tobacco smoking by prohibiting the use of billboards to advertise sugar-sweetened products and prohibiting the sponsorship of sporting activities by manufacturers and importers of drinks and foods [22]. There could also be a push to have sugar alternatives. Sugar-free drinks could be marketed over traditional sugar-laden drinks. The marketing campaign could include young people, such as sports stars, and community leaders. Having the sugar-free options available will allow for a massive reduction in total calories consumed.

Studies show that it is much easier to achieve significant health-related behavior changes, among children, both in eating and in physical activities [23]. Adult behavior is much more difficult to change, which is why community-based programs and social marketing campaigns advocating healthy eating have had limited success [23]. Social marketing by the government should show children consuming fresh local foods. The policy should focus on targeting children as “champions of health behavior changes” in Samoa, with baseline and monitoring studies by the Ministry of Health to assess whether the objectives are being achieved, over at least five to 10 years. For example, screening a healthy cooking segment on television using inexpensive healthy food would be advantageous. This could possibly spark change. Consideration should also be given to prohibiting children under 10 years old from buying any form of sugar-sweetened-product, unless the child is accompanied by an adult, as in the Tobacco Control Act [24]. This policy has been adopted by the state of Oaxaca in Mexico which banned the sale of soft drinks and high-calorie snack foods to children in 2020 [25]. Another measure could be to ban supermarkets from displaying sugar-sweetened products at the checkout points and the entrance to the supermarkets. Currently, in Samoa, sugar-sweetened products along with crisps and similar snack foods are strategically displayed on the racks either facing the entrance of the supermarkets, the checkout points, or on the checkout benches. A measure of this kind is to be implemented in the United Kingdom by mid-2022 to combat obesity [26]. Consumer education is needed so consumers understand the need to check food labeling and how to read them. A specific policy should require food labels to conform to international labeling standards. Food vendors should also be required to reveal the ingredients and energy value of the food they are offering in units such as grams or calories. In the United Kingdom, consumer education policies are now supported with instructions to restaurant owners to reduce the calories by 20% in popular takeaway foods.

To increase fresh local food production, since 2019 agriculture and fisheries project has aimed dependence on imported food and urbanization but seems to be making slow progress. In Samoa’s most populous region, the urbanizing Northwest Upolu, 34% of households were recorded as having no access to agricultural land compared to only 9.7% of households in rural Upolu and Savaii [27]. However limited land need not be a barrier, there are several examples in the vicinity of Apia town of small-scale commercial vegetable growers using growing tunnels on small sections of land next to their houses. Measures to encourage the production of fresh local food as a small business could include subsidies of stock feed and other agricultural inputs and, in particular, increasing extension activities for food production at the community level. Home gardening for improved household nutrition has long been promoted for women and youth, but there has so far been little emphasis on teaching ways to cook vegetables, especially those vegetables which can be easily grown in Samoa but which were not a traditional dietary item. There is also a need for government to increase the minimum wage so that the people can afford to buy fresh local food.

Urban and infrastructure planning also needs attention. In the rapidly growing urban areas around the town of Apia and Northwest Upolu, there are no playgrounds, sports fields, or gymnasiums, and no footpaths for walking and jogging. Large sports fields, a gymnasium and a swimming pool, adjacent to the town were built for international sports events and are not accessible to most Samoans [28]. A beautiful walking path around Apia harbour has been constructed, mainly with aid funds, and mainly for the enjoyments of tourists and urban elites, but few roads in populous areas have footpaths. The building of footpaths around the islands would be an example of how the government could make a change to benefit people living in rural and urban Samoa. In addition, like in neighbouring countries such as New Zealand and Australia, perhaps also to include the construction of bicycle lanes to encourage cycling (with helmets, etc. for safety) to and from school, to work, to church, and so forth. It is not only a visible form of exercise but also encouraging daily exercise, fresh air, less pollution, less congestion on the roads, less parking spaces needed, and less resource intensive. The churches should advocate for and acquire spaces for sports such as volleyball and other games adjacent to church halls. In workplaces, there should be morning exercises and regular breaks during working hours to encourage moving around and require warders to use the stairs instead of lifts and offer tokens to reward efforts and monitoring to identify resisters. Another issue that could be tackled in social marketing efforts could be to challenge the role model provided by mainly morbidly obese leaders in the community, workplace, church, government and communities, to combat the notion that there is a positive correlation between obesity, power and high social status [29].

## Conclusions

A much more vigorous multi-sector approach is needed, which would have to include politically unpopular policy measures aiming at addressing structural factors such as rapid urbanization, irresponsible social marketing of cheap unhealthy food items loaded with sugar, salt, and fat, by the food industry. There should be measures and strategies by the Government of Samoa that can easily and safely be integrated to encourage, enable and engage in daily physical activity targeting children, teenagers, and young adults. Without a much more vigorous and stringent multi-sectorial approach to address obesity in Samoa, progress seems unlikely. Government should provide change incentives that go deeper than just providing advisory messages urging the public to consume fresh local food and to exercise more. Addressing, as far as is possible, the structural forces driving obesity should become a much higher priority in national development planning by policymakers, and be supported by international development partners. Similar measures should be given more policy emphasis by the governments of other small Pacific Island states and territories.
